# Neuropsychological diagnosis of impaired mental functions in alcoholic disease of the second stage

**DOI:** 10.1192/j.eurpsy.2023.1949

**Published:** 2023-07-19

**Authors:** L. T. Baranskaya, E. I. Babushkina, V. I. Potapov

**Affiliations:** Psychiatry, Psychotherapy and Narcology, Ural State Medical University, Yekaterinburg, Russian Federation

## Abstract

**Introduction:**

In the global practice and in Russia, alcohol abuse in the population remains one of the main risk factors for disability and premature death of the able-bodied population

**Objectives:**

Systemic neuropsychological study of impaired mental functions caused by the toxic effects of alcohol.

**Methods:**

The study was in the substance use unit. Patients over the age of 24 years, with a period of abstinence from alcohol of at least 7 days before the study. The experimental group included 24 patients diagnosed with “Chronic alcoholism” stage II, (including 23 men and 1 woman). The age of patients was 47.1±4.5. The research method was standardized neuropsychological technique by A.R. Luria

**Results:**

In the study group, a direct relationship was found between disorders in the mental functions (frontal lobe of the brain) and the age of patients suffering from alcohol dependence (r = 0.477; p<0.05). This indicates premature cognitive aging, which manifested in a decrease in the processes of processing new information and working memory. With age, such patients become emotionally labile, impulsive, with behavioral disorders, similar to what occurs in different types of dementia and is the result of damage to the prefrontal lobe of the brain

**Conclusions:**

Because of the study, moderate and / or pronounced impaired of mental functions revealed in most patients: impaired of dynamic praxis in all processes deployed in the time; impaired of voluntary regulation of behavior and regulatory aspects of memory, attention, thinking, speech; impaired of orientation in space and in performing operations with spatial characteristics

**Disclosure of Interest:**

None Declared

